# Doxorubicin-induced nephrotoxicity: the protective role of a standardized ethanolic extract of *Andrographis paniculata* leaves

**DOI:** 10.3389/fphar.2025.1585965

**Published:** 2025-08-14

**Authors:** Wawaimuli Arozal, Oluebube Magnificient Eziefule, Septelia Inawati Wanandi, Melva Louisa, Syarifah Dewi, Nafrialdi, Nurjati Chairani Siregar, Ezekiel Makambwa

**Affiliations:** ^1^ Department of Pharmacology and Therapeutics, Faculty of Medicine, Universitas Indonesia, Jakarta, Indonesia; ^2^ Master’s Programme in Biomedical Sciences, Faculty of Medicine, Universitas Indonesia, Jakarta, Indonesia; ^3^ Department of Biochemistry and Molecular Biology, Faculty of Medicine, Universitas Indonesia, Jakarta, Indonesia; ^4^ Department of Pathology Anatomy, Faculty of Medicine, Universitas Indonesia, Jakarta, Indonesia; ^5^ Master’s Program in Pharmaceutical Sciences, Faculty of Pharmacy, Universitas Indonesia, Depok, Indonesia

**Keywords:** Andrographis paniculata, andrographolide, antioxidant, anti-inflammation, nephrotoxicity

## Abstract

**Introduction:**

Multi-organ toxicity, including nephrotoxicity, is a major drawback to the use of doxorubicin in chemotherapy. This study investigated the protective effect and possible mechanism of action of a standardized ethanolic extract of *Andrographis paniculata* (Burm.f.) Wall. ex Nees leaves (EEAP) capsule formula against doxorubicin (DOX)-induced nephrotoxicity.

**Method:**

DOX was administered intraperitoneally, while the EEAP capsule formula was given orally at doses of 125, 250, and 500 mg/kg BW. Kidney tissues were analyzed for concentrations of nuclear factor kappa B (NF-κB), superoxide dismutase (SOD), and total antioxidant capacity (TAC); mRNA expression levels of inflammatory markers, including nucleotide-binding oligomerization domain, leucine-rich repeat, and pyrin domain-containing protein 3 (NLRP3) and interleukin-1 beta (IL-1ß), were measured; plasma levels of kidney function parameters such as urea, creatinine, and electrolytes (sodium and calcium) were quantified. Histopathological changes were assessed using hematoxylin and eosin staining. Additionally, molecular docking was conducted to evaluate the interaction between andrographolide and the target proteins affected by DOX.

**Result:**

An increase in TAC concentration (*p* < 0.05), a decrease in NLRP3 mRNA expression (*p* < 0.05), and a reduction in serum sodium concentration (*p* < 0.05) were observed following EEAP administration. Minimal pathological alterations were noted in the cotreatment groups compared to the DOX-only group. Molecular docking revealed that andrographolide showed favorable binding energies with the target proteins (approximately -5 to -8 kcal/mol).

**Conclusion:**

It is suggested that EEAP conferred renal protection against DOX-induced damage primarily through the attenuation of oxidative stress and inflammation, with andrographolide playing a significant role in the observed protective effects.

## 1 Introduction

Doxorubicin (DOX) is a powerful chemotherapy drug, but its efficacy is hampered by tissue and organ toxicities, primarily affecting the heart, kidneys, and liver (Afsar et al., 2020; Burke et al., 1977; Renu et al., 2022). DOX’s ability to impair renal function is well-established, and for this reason, scientists routinely use DOX to create animal models of kidney failure for research purposes (Lee and Harris, 2011). As one of the excretory organs for DOX, the kidneys are directly exposed to the drug and its free radical byproducts. These free radicals can damage kidney cells, particularly the glomeruli and tubules (Afsar et al., 2020; Lee and Harris, 2011). Many studies have shown that the depletion of antioxidants like superoxide dismutase (SOD) and the upregulation of inflammatory proteins such as NF-κB, NLRP3, and IL-1β are associated with DOX-induced nephrotoxicity (Alasmari et al., 2022; Arunachalam et al., 2022; Wu et al., 2021). This suggests that investigating oxidative stress and inflammation could be a promising approach to mitigate DOX-induced nephrotoxicity.

The severity of chemotherapy-induced renal impairment depends on individual risk factors and how the kidneys handle the drug (Perazella and Moeckel, 2010). In the clinical setting, the current strategies for preventing chemotherapy-induced kidney toxicity include close monitoring of kidney function, encouraging increased fluid intake, adjusting chemotherapy doses, or even stopping the drug altogether (Santos et al., 2020). There is still a continuous search for better preventive alternatives for chemotherapy-induced renal toxicities. *Andrographis paniculata* (Burm.f.) Wall. ex Nees (*A. paniculata*) has shown potential in this regard and is being investigated (Adeoye et al., 2019; Singh et al., 2009).

*Andrographis paniculata*, also known as the king of bitters (Hossain et al., 2014), is an herbal plant belonging to the kingdom Plantae, division Angiospermae, and family Acanthaceae. Its genus and specie are *Andrographis* and *paniculata*, respectively (Bhaisare et al., 2023). The plant contains various compounds, including terpenes, flavonoids, and others like xanthones, noriridoids, and proteins. It is used in traditional medicine throughout many Asian countries, including China, Bangladesh, Malaysia, and Indonesia (Hossain et al., 2014). *A. paniculata* exhibits various pharmacological effects, including antioxidant, anti-inflammatory, anticancer, and renal protective effects (Adeoye et al., 2019; Hossain et al., 2014; Okhuarobo et al., 2014). Studies on *A. paniculata* have mainly utilized plant extracts or isolated bioactive compounds, with andrographolide being the most studied individual compound (Eziefule O. M. et al., 2024). Andrographolide, a diterpenoid lactone (a type of terpene), is considered the major bioactive compound in *A. paniculata* due to its broad range of pharmacological activities (Sareer et al., 2014). The anti-inflammatory and antioxidant properties of andrographolide have been extensively documented (Chang et al., 2014; Pasha et al., 2024; Shu et al., 2024; Yang et al., 2017). Shu et al. (2024) demonstrated that andrographolide attenuates oxidative stress by activating the Nrf2/HO-1 signaling pathway, leading to enhanced activity of endogenous antioxidant enzymes, including SOD, in mice model of ulcerative colitis. In the same study, pro-inflammatory cytokines such as interleukin-1β (IL-1β) were significantly suppressed (Shu et al., 2024). The inhibitory effect of andrographolide on NF-κB signaling is particularly noteworthy; numerous studies have identified andrographolide as a novel NF-κB inhibitor (Chang et al., 2014; Yang et al., 2017). In addition to direct inhibition (Pasha et al., 2024), andrographolide may also indirectly regulate NF-κB activity via the Nrf2/HO-1 axis (Shu et al., 2024). Heme oxygenase-1 (HO-1), a downstream target of Nrf2, is known to interfere with NF-κB activation (Shu et al., 2024), thereby attenuating the transcription of its pro-inflammatory downstream targets.

While andrographolide possesses proven pharmacological effects, isolating and administering it alone may be toxic in some cases. Research by Zhang et al. (2014) demonstrated that 26 patients who received andrographolide experienced acute kidney injury (AKI) (Zhang et al., 2014). Additionally, a phase 1 clinical trial of andrographolide was halted due to adverse events (Calabrese et al., 2000). Cai et al. (2025) recently demonstrated that andrographolide induces kidney injury and senescence by inhibiting the SIRT3/p53 signaling pathway. They further reported that disruption of the SIRT3–p53 interaction by andrographolide leads to increased acetylation of p53 and upregulation of its downstream target genes associated with inflammation, fibrosis, and senescence (Cai et al., 2025). To better harness the pharmacological effects of andrographolide with reduced toxicity, strategies such as administering the standardized plant extract, or modifying the andrographolide structure may become crucial (Dai et al., 2019). The use of standardized *A*. *paniculata* extract offers particular advantages as it contains additional bioactive constituents that may contribute to therapeutic efficacy (Chao and Lin, 2010). This combined action may yield a more synergistic and effective outcome, even at low doses. To the best of our knowledge, no published articles have reported toxicity associated with any *A. paniculata* extract. Studies in rodents have shown no observable toxicity even at high doses (5,000 mg/kg) (Worasuttayangkurn et al., 2019). Our previous study also demonstrated that the acute toxicity of the native extract used in this study was estimated to be greater than 2000 mg/kg (Arozal et al., 2024). These findings support the proposition that administering a standardized ethanolic extract of *A. paniculata*, rather than isolated andrographolide, may reduce the risk of toxicity while preserving or even enhancing therapeutic outcomes. Additionally, a study demonstrated that the bioavailability of andrographolide in the orally administered ethanolic extract of *A. paniculata* was four times higher than that of the orally administered andrographolide in rats (Chen et al., 2014). This adds further credence to administering the extract rather than andrographolide alone. Further, Adeoye et al. (2019) have shown that *A. paniculata* ethanolic extracts can protect against cisplatin-induced nephrotoxicity (Adeoye et al., 2019), but their effects on DOX-induced nephrotoxicity have not been extensively investigated.

This study aimed to investigate the protective effects and possible mechanism of action of a standardized ethanolic leaf extract of *A. paniculata* against DOX-induced renal toxicity. The antioxidant and anti-inflammatory effects of the extract, along with its impact on renal function and histopathology, were evaluated. Additionally, as andrographolide had been previously identified and characterized in this extract (Arozal et al., 2024), molecular docking was performed to investigate potential interactions between andrographolide and the proteins of interest. By elucidating the mechanisms underlying DOX-induced nephrotoxicity and the potential protective effects of EEAP, it is hoped that novel strategies for preventing or mitigating the detrimental side effects of chemotherapy can be developed.

## 2 Methods

### 2.1 Plant materials

The plant leaves were gathered by the staff from PT. Konimex, a pharmaceutical company in Indonesia, in April 2022 in Tawamangu, Central Java, Indonesia. The voucher specimen (ANPS-05) has been archived at the Faculty of Pharmacy, Universitas Gadjah Mada, Yogyakarta, Indonesia. The details for making the plant extract and analysis of andrographolide content have been published in our previous report (Arozal et al., 2024). Briefly, the extract was obtained by crushing dried leaves, soaking them in 90% ethanol, evaporating the solvent at 60°C under vacuum, drying the concentrate with a fluid bed granulator, and encapsulating it into 125 mg capsules containing 60 mg AP extract. The andrographolide content in the 60 mg AP extract (standardized) was measured to be 8.98% (5.39 mg) by HPLC fingerprinting.

### 2.2 Animal experimentation

Thirty male Sprague-Dawley rats (6–8 weeks old, 150–200 g) were obtained from Indonesia’s National Agency of Drug and Food Control specifically for this experiment. The rats were housed in a controlled environment (21°C, 55% humidity, 12-h light/dark cycle) with free access to food and water. This study upheld the ARRIVE guidelines, followed ethical guidelines for animal treatment, and was approved by the Ethics Committee of the Faculty of Medicine, University of Indonesia, on 8 August 2022. (document number KET-822/UN2.F1/ETIK/PPM.00.02/2022).

The rats were acclimatized for 2 weeks and were then randomly divided into five groups of six animals each. The groups received the following treatments for 4 weeks:

1. Group 1 (Normal): Received saline solution (0.9% NaCl) injected intraperitoneally (i.p.) once a week. This group served as the baseline for comparison.
2. Group 2 (DOX): Received doxorubicin (DOX) at 4 mg/kg body weight (BW) weekly via intraperitoneal injection, for a total dose of 16 mg/kg BW. This dose was used in a previous study (Arozal et al., 2024). DOX was obtained from Kalbe Pharma (Indonesia) as doxorubicin hydrochloride (2 mg/mL). The batch number and expiry date are VDXBA00026 and October 2022, respectively.
3. 3. Groups 3–5 (DOX + Extract): Received the same dose of DOX as Group 2 and were additionally administered the ethanolic extract of *Andrographis paniculata* (EEAP) orally at varying doses. Specifically, Groups 3, 4, and 5 received 125, 250, and 500 mg/kg BW/day of the extract capsule formulation, corresponding to 60, 120, and 240 mg/kg BW/day of EEAP, respectively.

#### 2.2.1 Method of euthanasia for animal experiment

Rats were administered ketamine (80 mg/kg body weight) and xylazine (8.0 mg/kg body weight) via intraperitoneal injection to induce unconsciousness and alleviate the pain associated with cardiac puncture for blood collection. Unconsciousness was confirmed by the animals’ lack of response to a toe pinch. It is important to note that during the animal experimentation, no other procedures that could cause pain or distress were performed on the rats aside from the intraperitoneal injections.

### 2.3 Serum and tissue preparation

Blood and kidney tissue collection followed established protocols at the faculty’s animal facility. Blood samples were obtained by puncturing the heart and then centrifuged at 3,000 *g* for 10 min to isolate the serum for measuring electrolytes, urea, and creatinine. Following euthanasia, the kidneys were removed and rinsed with a chilled saline solution. The left kidney was fixed in 10% neutral buffered formalin for 48 h. To prepare for microscopic examination, the specimens were dehydrated through a series of graded alcohols, cleared with xylene, and then embedded in paraffin wax for hardening. Finally, the paraffin blocks were sectioned into 5-μm slices using a microtome and stained with hematoxylin-eosin (H&E) for analysis. The right kidney was stored at −80°C for qRT-PCR, ELISA, and other biochemical assays. For RNA isolation, a portion of the right kidney was homogenized using Trizol buffer at a ratio of 1:20 (w/v). For ELISA and other biochemical assays, another portion of the tissue was homogenized with PBS in a ratio of 1:9 (w/v) on ice.

### 2.4 RNA isolation

Following tissue/cell lysis to release cellular components, chloroform (20% volume) was added. The mixture was then centrifuged at 12,000 rpm for 15 min at 4°C, separating into three phases. The colorless upper aqueous phase, containing RNA, was carefully collected. Next, ethyl alcohol (95%–100% of sample volume) was added to the aqueous phase and mixed. The mixture was transferred to a Zymo-Spin IIICG column and centrifuged for 1 min at 12,000 rpm and 4°C. The flow-through was discarded. RNA purification continued using the Zymo-Spin IIICG column. Briefly, 400 μL Direct-zol RNA PreWash was added to the column, followed by centrifugation (1 min, 12,000 rpm, 4°C). The flow-through was discarded, and this wash step was repeated once. Subsequently, 700 μL RNA Wash Buffer was added and centrifuged (1 min, 12,000 rpm, 4°C) to remove residual wash buffer. The column was then transferred to an RNase-free tube. Finally, 50 μL DNase/RNase-Free Water was added directly to the column matrix and centrifuged (1 min, 12,000 rpm, 4°C) to elute the purified RNA. RNA concentration was determined spectrophotometrically (Varioskan flash) at a wavelength of 260 nm and purity by measuring the 260/280 absorbance ratio. 1.8 was considered indicative of pure RNA yield.

### 2.5 cDNA synthesis, qRT-PCR, and mRNA expression

Only RNA samples meeting a pre-defined purity threshold were used for subsequent cDNA synthesis. This process was carried out using the ReverTra Ace^®^ qPCR RT Master Mix (Toyobo BioTech, Osaka, Japan) according to the manufacturer’s instructions. The concentration and purity of the resulting cDNA were also evaluated spectrophotometrically at a wavelength of 260 nm. Established quantitative real-time PCR (qRT-PCR) methods were employed for gene amplification. The chosen kit was the SensiFAST™ SYBR^®^ No-ROX kit mix (Meridian Bioscience, Cincinnati, Ohio, United States). A uniform thermal cycling program was applied to all three genes of interest, including the reference gene. This program consisted of denaturation at 95°C, annealing at 60°C, and extension at 72°C. The specific genes and their sequences used in this study were the same as those in our previous study (Eziefule OM. et al., 2024). Gene amplification was performed to obtain cycle threshold (Ct) values. These Ct values were then used with the Livak method to determine the relative expression levels of the target genes.

### 2.6 Analysis of the concentration of NF-кB and oxidative stress parameters

NF-κB levels in kidney tissue were determined using an ELISA kit (Catalog No. BZ-22183961-EB; Bioenzy, Jakarta, Indonesia). SOD activity and total antioxidant capacity were determined in the kidney tissue using colorimetric assay kits (Catalog Nos. DG-SOD400 and DG-TAC200, respectively) from DoGenBio Co., Ltd., Korea.

### 2.7 Analysis of kidney function

The serum concentrations of sodium and calcium ions were determined using colorimetric assay kits from Elabscience (Catalog No: E-BC-K207-M for sodium and E-BC-K103-M for calcium). The Jaffe method (Husdan and Rapoport, 1968) was employed to quantify serum creatinine, and the diacetyl monoxime method (Foster and Hochholzer, 1971) to quantify blood urea nitrogen (BUN) levels.

### 2.8 Histopathological analysis of kidney tissues

Histological slides stained with H&E were analyzed using light microscopy to assess changes in kidney tissue histopathology. To maintain objectivity, two pathologists, who were unaware of the experimental groups, evaluated the slides based on a modified scoring criterion from a previous study (Refaie et al., 2016).

Four slides from each group were selected for examination by the pathologists, who then reviewed them to determine the appropriate scoring system. On each slide, researchers randomly captured 5 non-overlapping fields at ×40 magnification. A semi-quantitative scoring system assessed the frequency and severity of specific kidney lesions. Normal (score–): No histological alterations observed; very mild (score+): Lesions present in a limited number of fields, somewhere between normal and mild; mild (score++): Lesions affecting less than 25% of the examined fields. Moderate (score+++): Lesions present in less than 50% of the examined fields. Severe (score++++): Lesions affecting 50% and above of the examined fields.

### 2.9 Hardware and software

The *in silico* study was conducted on a Lenovo Legion T5 26IAB7 (LAB-KOMB-03) 12th Gen Intel(R) Core™ i7-12700 with a 2.1 GHz CPU, 128GB RAM, and a 64-bit Windows 11 Version 22H2 operating system. UCSF Chimera X (version 1.6.1), UCSF Chimera (version 1.17.3) AutoDockTools (version 1.5.7), and Avogadro (version 1.2.0) were used for data preparation. Whilst AutoDock Vina was used on PyRx (version 1.1) for the molecular docking. BioVia’s Discovery Studio Visualizer (version 24.1.0.23298) was used for the visualization of molecular interactions.

### 2.10 Data acquisition and preparation

A search of the targets using their names on UniProt (https://www.uniprot.org/) gave the best 3D structures which were then retrieved from the Protein Data Bank (https://www.rcsb.org/) in pdb format. For the reference ligands, an open search on Google and other databases was conducted using related keywords. Vanillyl alcohol (VA) was found to be one of the most studied ligands for p65 (Zlatanova et al., 2024) whereas Dapansutrile (Kiran et al., 2022) was selected as the reference ligand for the NPR3-NACHT domain. MLN120B is a selective IKKβ inhibitor that directly binds to the kinase domain of IKKβ (Wen et al., 2006). SOD is a special case, as it is the only target that is expected to be activated by its ligand. Iodoacetic acid (IAA) is one of the small molecules that was found to act as an agonist when binding to SOD (Wang et al., 2019). All structures of the ligands were retrieved from the PubChem database (https://pubchem.ncbi.nlm.nih.gov/) as sdf files except for (S)-2. The reference compound’s structure for the target protein IL-1β (S-2) (Hommel et al., 2023) was modeled using ChemDraw Ultra (version 12.0.2.1076), as its structure wasn’t found in the PubChem database. The following criteria were used for selecting protein structures; *homo sapiens*, high resolution, completeness of the protein, and availability as a protein-ligand complex. The structures with PDB IDs 1NFL,7ALV, and 3BRV were missing residues and atoms which were filled using the Swiss model (https://swissmodel.expasy.org/) (Waterhouse et al., 2018; Zubair et al., 2023). All retrieved protein structures were stripped of heteroatoms, sidechains, and water using AutoDockTools, and then polar hydrogen atoms and Kollman charges were added to them and saved as pdbqt files. All ligands were run through Avogadro for geometry optimization using the mmff94 forcefield on the steepest gradient algorithm with 500 steps and saved as mol2 files.

### 2.11 Molecular docking

Computed Atlas of Surface Topography of proteins (Castp) (http://cast.engr.uic.edu) was used together with UCSF Chimera for the binding pocket analysis of the proteins p65, IKKβ, and SOD (Tian et al., 2018). The active site of SOD was predicted in previous studies (Song et al., 2023; Wang et al., 2019). Subsequently, the active sites of IL-1β were also acquired from an earlier study (Liu et al., 2023), whereas the binding site of the native 7ALV ligand was used as the active site of NLRP3 (Dekker et al., 2021). Using the defined binding sites (Table 1) for the grid box, molecular docking was carried out on AutoDock Vina for all target proteins against Andrographolide, Doxorubicin, Doxorubicinol, and the reference ligand using the parameters: exhaustiveness set to 8 and number of modes set to 50 (Trott and Olson, 2010).

**TABLE 1:** Selected protein targets and their binding sites.

Target protein	PDB ID	Reference ligand	Binding site: (Centre) (Points Å)
NF-кB (relA p65)	1NFL	Vanillyl alcohol	(4.28; 47.31; 16.19) (30.83; 44.23; 33.27)
NLRP3-NACHT domain	7ALV	Dapansutrile	(15.87; 35.15; 127.53) (19.73; 24.53; 22.87)
IL-1β	8C3U	(S)-2	(-3.78; −23.51; −20.27) (40.45; 26.46; 33.66)
IKKβ	3BRV	MLN120B	(-18.74; −13.07; 13.30) (15.05; 28.61; 18.89)
SOD	1AZV	IAA	(48.27; 47.97; 11.59) (30.77; 32.10; 38.31)

### 2.12 Statistical analysis

The results, except histopathology data, were presented as an average value (mean) with an indicator of variability (standard error of the mean (SEM)). To compare the groups, a one-way analysis of variance (ANOVA) was employed. This was followed by Tukey’s *post hoc* test to pinpoint specific group differences. All analyses except for histopathology data were performed using GraphPad Prism software (version 8.0). A p-value of less than 0.05 was considered statistically significant. A semi-quantitative scoring system was employed for the histopathological analysis.

## 3 Results

### 3.1 Effect of EEAP on oxidative stress parameters

**FIGURE 1:**
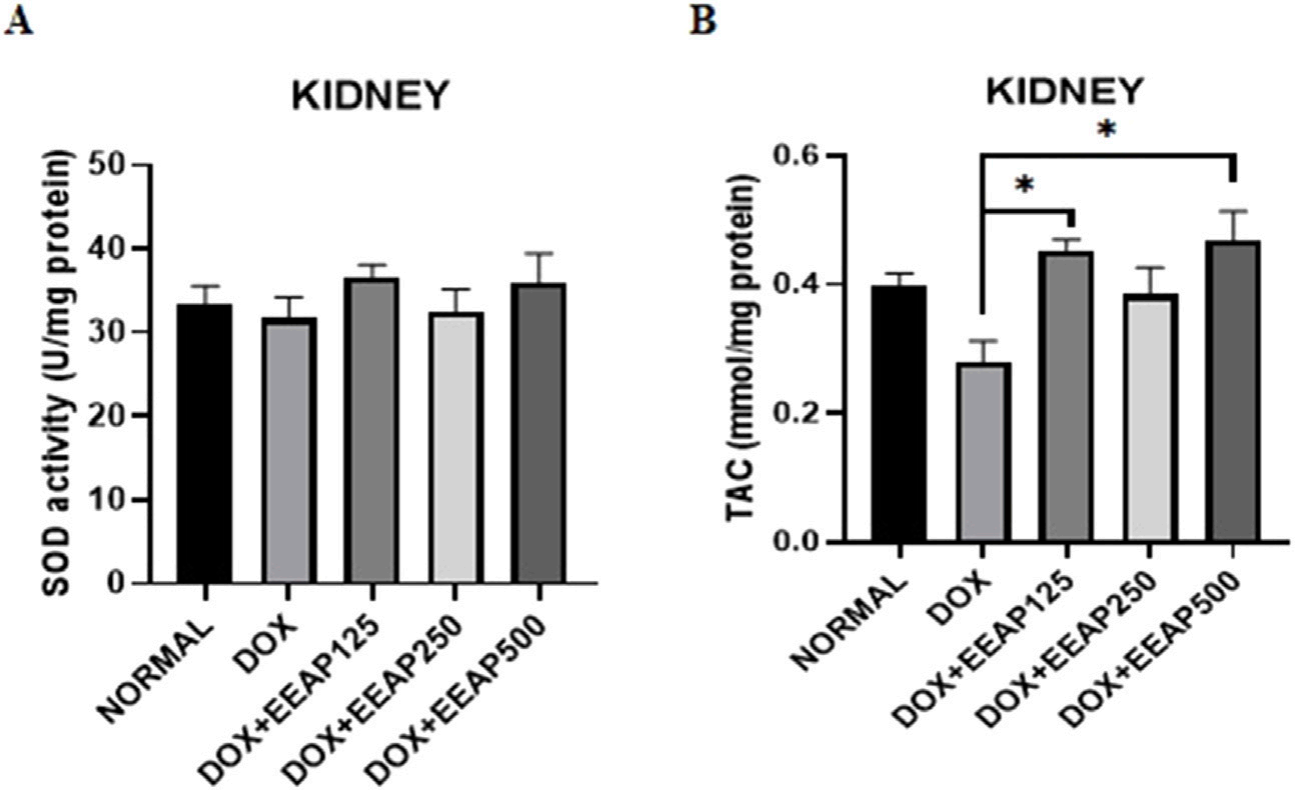
The effect of EEAP on oxidative stress parameters in kidney tissues of DOX-treated rats. **(A)** depicts SOD activity in U per mg protein, with no significant differences across groups. **(B)** shows TAC in mmol per mg protein, with significant differences indicated by asterisks. The values were presented as Mean ± SEM (*p < 0.05). Abbreviations: DOX, doxorubicin; EEAP, Ethanolic extract of *Andrographis paniculata leaves*; SOD, Superoxide dismutase; TAC, Total antioxidant capacity; DOX + EEAP125, Doxorubicin + ethanolic extract of *Andrographis paniculata* leaves capsule formula 125 mg/kg BW; DOX + EEAP250, Doxorubicin + ethanolic extract of *Andrographis paniculata* leaves capsule formula 250 mg/kg BW; DOX + EEAP500, Doxorubicin + ethanolic extract of *Andrographis paniculata* leaves capsule formula 500 mg/kg BW; SEM, Standard error mean.

The result of SOD activity and total antioxidant capacity (TAC) in kidney tissues are presented in Figure 1. Figure 1A showed no statistically significant result between all groups. The SOD activity in the DOX-only group in comparison with the normal group decreased by 5% (33.44 ± 2.11 vs. 31.69 ± 2.49 (Normal vs. DOX)). In comparison to the DOX-only group, the SOD activity in the co-treatment groups DOX + EEAP125, DOX + EEAP250, and DOX + EEAP500 increased by 15.43% (31.69 ± 2.49 vs. 36.58 ± 1.47 (DOX vs. DOX + EEAP125) 2.7% (31.69 ± 2.49 vs. 32.56 ± 2.63) and 13.54% (31.69 ± 2.9 vs. 35.98 ± 3.48) respectively.

Figure 1B showed statistically significant results between the DOX group and two co-treatment groups (DOX + EEAP125 and DOX + EEAP500). The TAC concentration in the DOX-only group compared to the normal group decreased by 28.20% (0.39 ± 0.02 vs. 0.28 ± 0.03 (Normal vs. DOX)). The TAC concentration in the DOX + EEAP125, DOX + EEAP250, and DOX + EEAP500 compared to the DOX-only group increased by 60.71% (0.28 ± 0.03 vs. 0.45 ± 0.02, p < 0.05 (DOX vs. DOX + EEAP125)), 35.71% (0.28 ± 0.03 vs. 0.38 ± 0.04 (DOX vs. EEAP250)), and 67.86% (0.28 ± 0.03 vs. 0.47 ± 0.04, p < 0.05 (DOX vs. EEAP500)), respectively.

### 3.2 Effect of EEAP on inflammatory parameters

**FIGURE 2:**
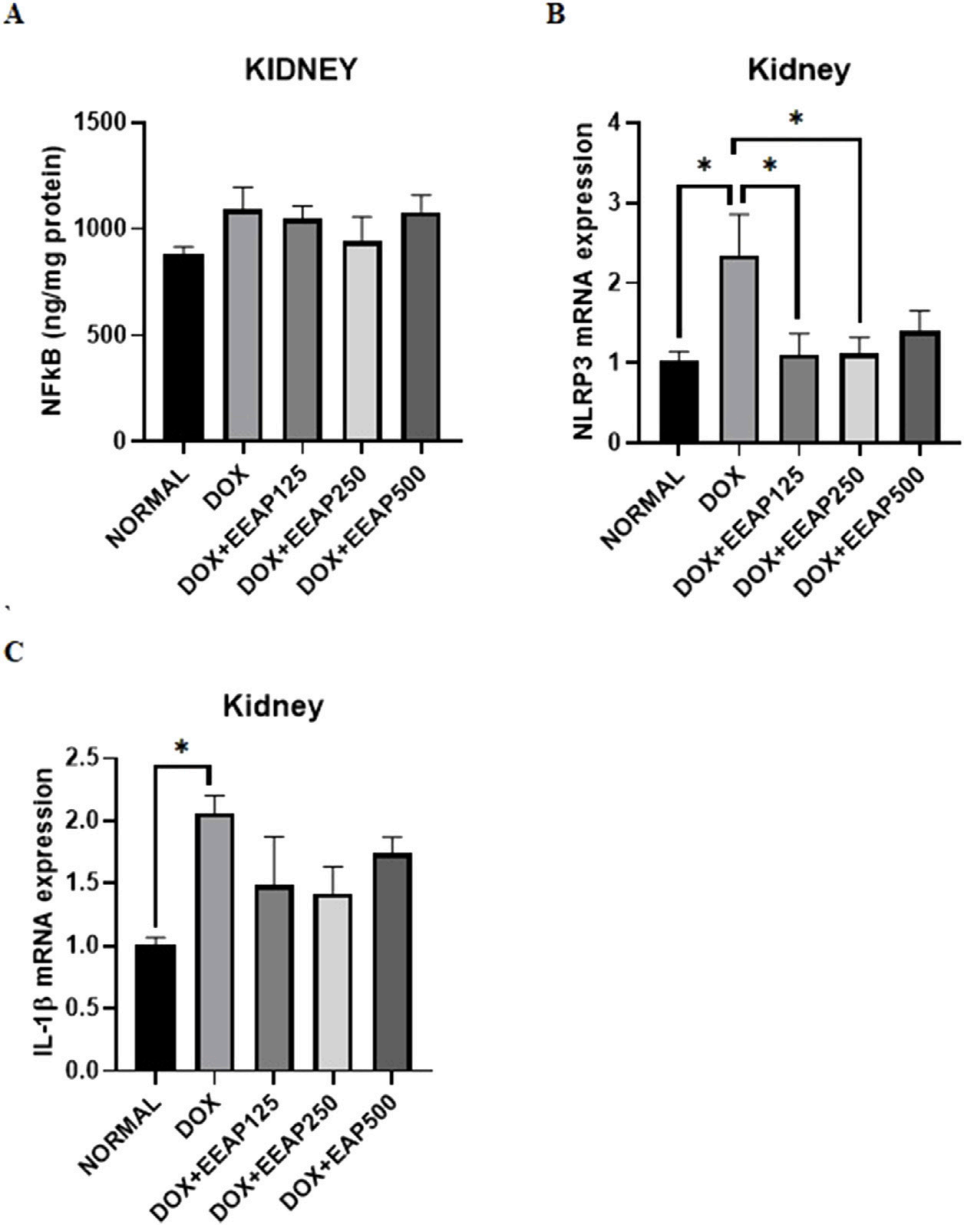
The effect of EEAP on inflammatory parameters in kidney tissues of DOX-treated rats. **(A)** displays NFkB levels with no significant differences across groups. **(B)** shows NLRP3 mRNA expression, peaking in the DOX group. **(C)** highlights IL-1β mRNA expression, also the highest in the DOX group. Asterisks indicate statistical significance. The values were presented as Mean ± SEM (*p < 0.05). Abbreviations: DOX, doxorubicin; EEAP, Ethanolic extract of *Andrographis paniculata leaves*; NFкB, Nuclear factor kappa-light-chain-enhancer of activated B cells; NLRP3, nucleotide-binding domain, leucine-rich-containing family, pyrin domain-containing-3; DOX + EEAP125, Doxorubicin + ethanolic extract of *Andrographis paniculata* leaves capsule formula 125 mg/kg BW; DOX + EEAP250, Doxorubicin + ethanolic extract of *Andrographis paniculata* leaves capsule formula 250 mg/kg BW; DOX + EEAP500, Doxorubicin + ethanolic extract of *Andrographis paniculata* leaves capsule formula 500 mg/kg BW; SEM, Standard error mean.

The results of NFкB concentration, NLRP3, and IL-1β mRNA expressions in kidney tissues are presented in Figure 2. Figure 2A showed no statistically significant result between all groups. The NFкB concentration in the DOX-only group in comparison with the normal group increased by 23.99% (882.60 ± 34.65 vs. 1,094.41 ± 102.5 (Normal vs. DOX)). In comparison to the DOX-only group, the NFкB concentration in the co-treatment groups DOX + EEAP125, DOX + EEAP250, and DOX + EEAP500 decreased by 4.31% (1,094.41 ± 102.5 vs. 1,047.20 ± 61.48 (DOX vs. DOX + EEAP125)), 13.69% (1,094.41 ± 102.5 vs. 944.523 ± 114 (DOX vs. DOX + EEAP250)) and 1.23% (1,094.41 ± 102.5 vs. 1,080.92 ± 77.85 (DOX vs. DOX + EEAP500)) respectively.

Figure 2B showed statistically significant results between the normal group and the DOX-only group and between the DOX-only group and two co-treatment groups (DOX + EEAP125 and DOX + EEAP250). The mRNA expression of NLRP3 in the DOX-only group compared to the normal group increased by 128.16% (1.03 ± 0.11 vs. 2.35 ± 0.51, *p <* 0.05 (Normal vs. DOX)). NLRP3 mRNA expression in DOX + EEAP125, DOX + EEAP250, and DOX + EEAP500 compared to the DOX-only group decreased by 52.78% (2.35 ± 0.51 vs. 1.11 ± 0.26, *p <* 0.05 (DOX vs. DOX + EEAP125)), 51.91% (2.35 ± 0.51 vs. 1.13 ± 0.19, *p <* 0.05 (DOX vs. EEAP250)), and 40.43% (2.35 ± 0.51 vs. 1.43 ± 0.25 (DOX vs. EEAP500)), respectively.

Lastly, Figure 2C showed statistical significance only between the normal group and the DOX-only group. The mRNA expression of IL-1β in the DOX-only group in comparison to the normal group increased by 103.96% (1.01 ± 0.06 vs. 2.06 ± 0.14, *p <* 0.05 (Normal vs. DOX)). IL-1β mRNA expression in DOX + EEAP125, DOX + EEAP250, and DOX + EEAP500 compared to the DOX-only group decreased by 28.16% (2.06 ± 0.14 vs. 1.48 ± 0.39 (DOX vs. DOX + EEAP125)), 31.07% (2.06 ± 0.14 vs. 1.42 ± 0.21 (DOX vs. EEAP250)), and 15.53% (2.06 ± 0.14 vs. 1.74 ± 0.13 (DOX vs. EEAP500)), respectively.

### 3.3 Effect of EEAP on renal function markers

**FIGURE 3:**
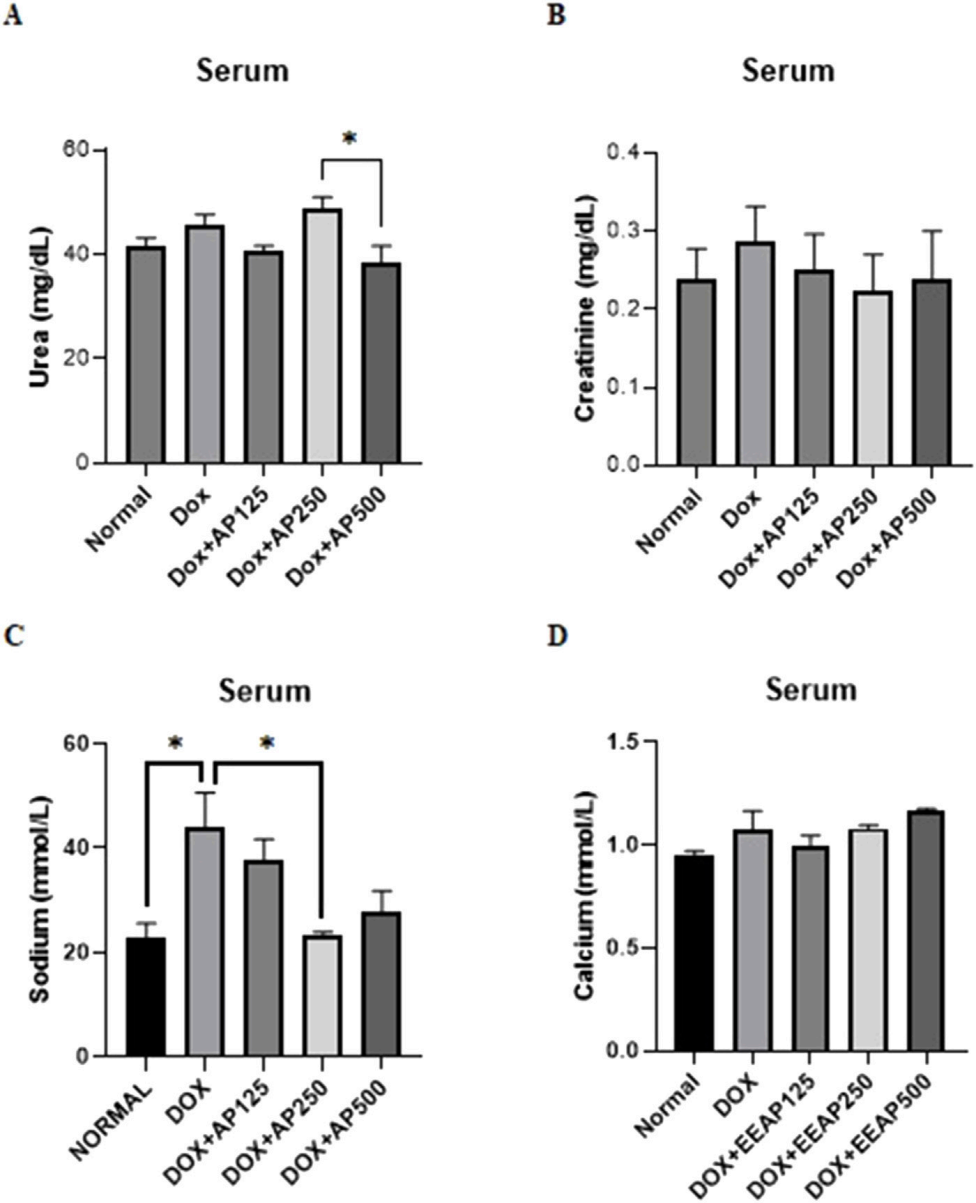
The effect of EEAP on renal function markers in serum of DOX-treated rats. **(A)** shows urea levels. **(B)** measures creatinine, showing no significant differences. **(C)** displays sodium, with an elevated level in DOX group. **(D)** shows calcium levels, with no substantial variations across groups. The values were presented as Mean ± SEM (*p < 0.05). Abbreviations: DOX, doxorubicin; EEAP, Ethanolic extract of *Andrographis paniculata leaves*; DOX + EEAP125, Doxorubicin + ethanolic extract of *Andrographis paniculata* leaves capsule formula 125 mg/kg BW; DOX + EEAP250, Doxorubicin + ethanolic extract of *Andrographis paniculata* leaves capsule formula 250 mg/kg BW; DOX + EEAP500, Doxorubicin + ethanolic extract of *Andrographis paniculata* leaves capsule formula 500 mg/kg BW; SEM, Standard error mean.

The results of urea, creatinine, sodium, and calcium levels in serum are presented in Figure 3. Figure 3A showed statistical significance only between two cotreatment groups (DOX + EEAP250 and DOX + EEAP500). The urea level in the DOX-only group in comparison with the normal group increased by 9.98% (41.67 ± 1.52 vs. 45.83 ± 1.86 (Normal vs. DOX)). In comparison to the DOX-only group, the urea level in the co-treatment groups DOX + EEAP125 and DOX + EEAP500 decreased by 10.89% (45.83 ± 1.86 vs. 40.84 ± 0.83 (DOX vs. DOX + EEAP125)), and 15.91% (45.83 ± 1.86 vs. 38.54 ± 3.13 (DOX vs. DOX + EEAP500)) respectively and DOX + EEAP250 increased by 6.83% (45.83 ± 1.86 vs. 48.96 ± 1.99 (DOX vs. DOX + EEAP250)).

Figure 3B showed no statistically significant result between all groups. The creatinine serum level in the DOX-only group compared to the normal group increased by 20.83% (0.24 ± 0.04 vs. 0.29 ± 0.04 (Normal vs. DOX)). Creatinine serum levels in DOX + EEAP125, DOX + EEAP250, and DOX + EEAP500 compared to the DOX-only group decreased by 13.79% (0.29 ± 0.04 vs. 0.25 ± 0.04 (DOX vs. DOX + EEAP125)), 20.69% (0.29 ± 0.04 vs. 0.23 ± 0.05 (DOX vs. EEAP250)), and 17.24% (0.29 ± 0.04 vs. 0.24 ± 0.06 (DOX vs. EEAP500)), respectively.

Further, Figure 3C showed statistical significance between the normal and DOX-only groups and between the DOX-only and DOX + EEAP250 groups. The sodium concentration in the DOX-only group compared to the normal group increased by 93.22% (22.71 ± 2.86 vs. 43.88 ± 6.77, *p <* 0.05 (Normal vs. DOX)). The sodium concentration in the DOX + EEAP125, DOX + EEAP250, and DOX + EEAP500 compared to the DOX-only group decreased by 14.54% (43.88 ± 6.77 vs. 37.50 ± 4.18 (DOX vs. DOX + EEAP125)), 46.90% (43.88 ± 6.77 vs. 23.30 ± 0.61, *p <* 0.05 (DOX vs. EEAP250)), and 37.01% (43.88 ± 6.77 vs. 27.64 ± 4.198 (DOX vs. EEAP500)), respectively.

Lastly, Figure 3D showed no statistically significant result between all groups. The calcium serum concentration in the DOX-only group compared to the normal group increased by 12.63% (0.95 ± 0.02 vs. 1.07 ± 0.09 (Normal vs. DOX)). Calcium serum concentration in DOX + EEAP125, compared to the DOX-only group, decreased by 6.54% (1.07 ± 0.09 vs. 1.00 ± 0.05 (DOX vs. DOX + EEAP125)). The DOX + EEAP250 and DOX + EEAP500 groups, in comparison to the DOX-only group, increased by 0.93% (1.07 ± 0.09 vs. 1.08 ± 0.02 (DOX vs. EEAP250)), and 8.41% (1.07 ± 0.09 vs. 1.16 ± 0.01 (DOX vs. EEAP500)), respectively.

### 3.4 Effect of EEAP on histopathological features

**FIGURE 4:**
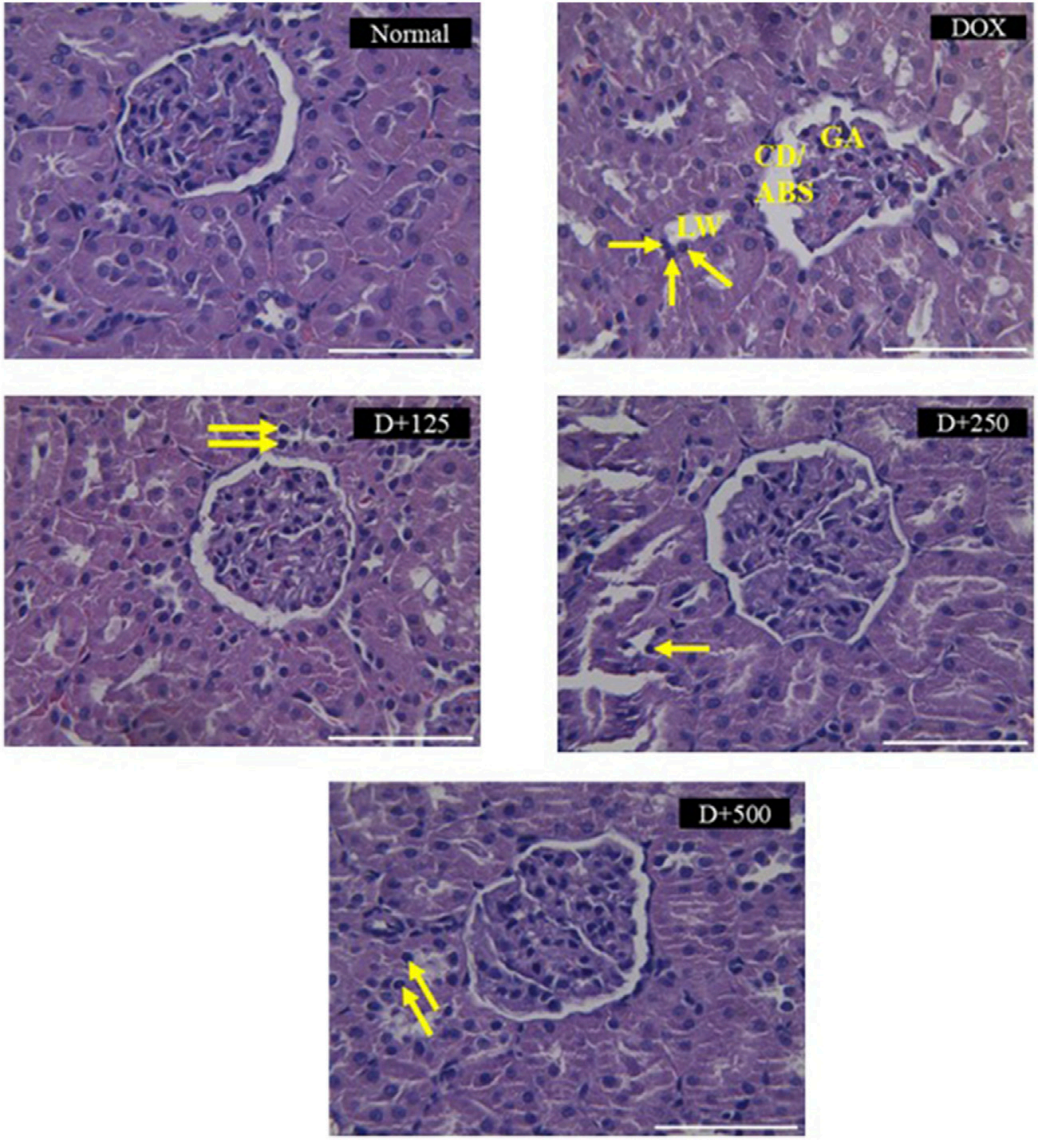
Histopathological changes induced by doxorubicin in rat kidney tissues stained with H&E and the protective effects of EEAP against these alterations. A semi-quantitative scoring system was employed for the histopathological analysis. Magnification was ×40. Normal: Normal group renal section exhibiting normal histology. DOX: DOX-only group renal section exhibited degenerative changes, capsule distortion, abnormal Bowman’s space, glomerular atrophy, tubular lumen widening, and pyknosis. D+125: DOX + EEAP125 group exhibited very mild and mild histopathological changes. D+250: DOX + EEAP250 group exhibited very mild and mild histopathological changes. D+500: The DOX + EEAP500 group exhibited mild histopathological changes. CD, capsule distortion; ABS, abnormal Bowman’s space; GA, glomerular atrophy; LW, lumen widening. Pyknosis is indicated by the arrows in the images. DOX + EEAP125, Doxorubicin + ethanolic extract of *Andrographis paniculata* leaves capsule formula 125 mg/kg BW; DOX + EEAP250, Doxorubicin + ethanolic extract of *Andrographis paniculata* leaves capsule formula 250 mg/kg BW; DOX + EEAP500, Doxorubicin + ethanolic extract of *Andrographis paniculata* leaves capsule formula 500 mg/kg BW.

Figure 4 illustrates the effect of EEAP on DOX-induced histopathology changes in rat kidney tissues stained with H&E. DOX treatment induced changes like capsule distortion, abnormal Bowman’s space, glomerular atrophy, tubular lumen widening, and pyknosis. The normal group maintained normal renal tissue histology. Co-treatment groups showed reduced pathological features. (Table 2).

**TABLE 2:** The result of renal tissue scoring.

Findings	Normal	DOX	DOX + EEAP125	DOX + EEAP250	DOX + EEAP500
Capsule distortion/abnormal bowman’s space	-	+++	**+**	**+**	++
Glomerular atrophy	-	+++	**+**	**+**	++
Tubular lumen widening	-	+++	++	++	++
Pyknosis	**+**	+++	++	++	++

### 3.5 Binding sites of target proteins

Table 1 shows the binding sites of protein targets.

### 3.6 Binding pocket analysis

**FIGURE 5:**
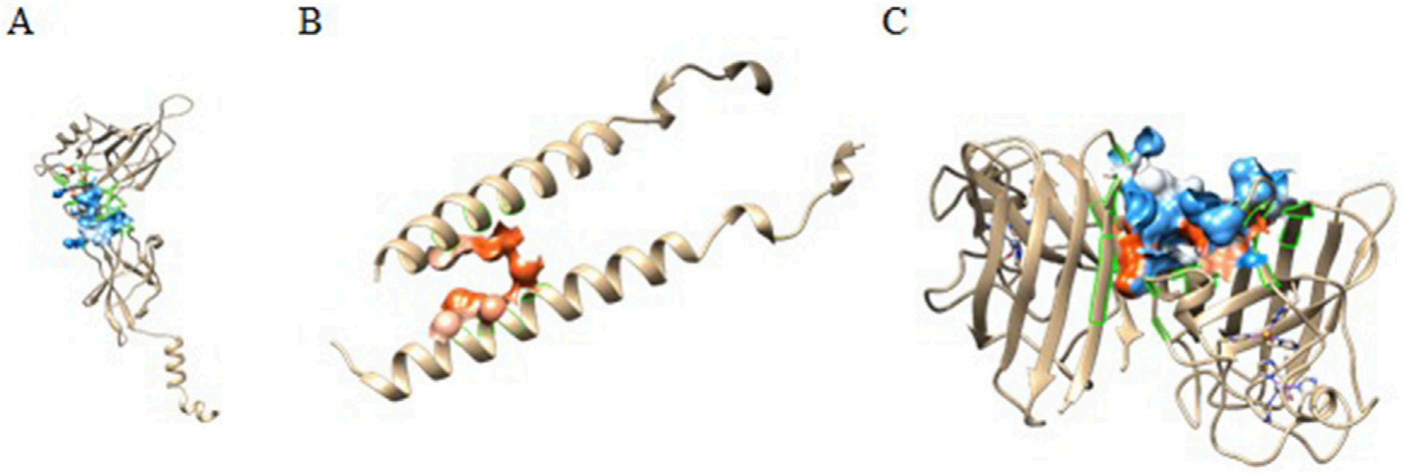
CastP predicted active sites of **(A)** P65, **(B)** IKKβ, and **(C)** SOD.

Castp provided several pockets for the analyzed protein structures. Figure 5 shows the chosen pockets on their respective structures. The program uses different parameters to rank binding pockets, including the Area of the pocket, its volume, and the number of openings at the pocket. In this study, the pockets in Table 3 were chosen as active sites.

**TABLE 3:** Binding pocket details of the chosen pockets predicted by Castp.

Protein	Pocket Id	Area (Å^2^)	Volume (Å^3^)	Openings
P65	2	508.3	737.7	2
IKKβ	3	76.3	198.8	1
SOD	1	701	1,247.5	6

### 3.7 Binding affinity

Autodock Vina uses a hybrid scoring function, which enhances its abilities to accurately predict the binding affinity between a receptor’s active site and a ligand molecule (Trott and Olson, 2010). It is said to be faster and more accurate than AutoDock 4, especially when used to dock targets like kinases (Vieira and Sousa, 2019). In this study, generally good binding energies (Table 4) were observed for all protein-ligand interactions, though doxorubicin exhibited better binding than all other ligands in most interactions. Most of the reference ligands exhibited poor binding energies as compared to andrographolide. Particularly, for the targets NLRP3-NACHT domain and SOD, the binding energy of andrographolide is around 4 magnitudes better than that of the reference. With regards to these targets, the binding energies of doxorubicin and andrographolide are comparable, though doxorubicin has a 1-2 magnitude better binding energy.

**TABLE 4:** Lowest binding energies of the ligands to the targets.

Target	Doxorubicin (Kcal/mol)	Doxorubicinol (Kcal/mol)	Reference ligand (Kcal/mol)	Andrographolide (Kcal/mol)
NF-кB (rel 65)	−7.833	−7.455	−4.805	−6.951
NLRP3-NACHT domain	−9.435	−8.989	−4.529	−8.223
IL-1β	−7.536	−7.276	−9.580	−7.616
IKKβ	−5.425	−5.652	−6.982	−5.441
SOD	−9.491	−9.346	−3.505	−7.578

### 3.8 Binding interactions

To get a better understanding of the suitability of a molecule to act as a drug molecule, it is important to understand the type and quality of interactions it makes with the active site of the target macromolecule (Table 5). A lot of factors are responsible for a strong bond that might lead to a more stable complex. Most studies focus on the types of interaction and their distances. The most common strong interactions include hydrogen bonds and hydrophobic interactions.

**TABLE 5:** Interactions with distances less than 5 Å between targets and listed ligands by PLIP (Adasme et al., 2021). Rs is the Residue involved in the interaction, IT is the interaction type, Ds is the Distance in Angstroms, Hp is the hydrophobic interaction, HD is the hydrogen donor, and HA is the hydrogen acceptor.

Target		Doxorubicin		Doxorubicinol		Reference ligand		Andrographolide	
	IT	Rs	Ds (Å)	Rs	Ds (Å)	Rs	Ds (Å)	Rs	Ds (Å)
NF-кB (Chain A)	Hp	PRO 28 PRO 28	3.47 3.84	PRO28	3.80	ASP258	3.66	LYS202 LYS202 LYS202 VAL225 GLN228	3.84 3.53 3.62 3.58 3.81
	HD	LYS9 ARG16 ARG16 GLY25 SER26 SER32 LYS37 HIS39	3.50 2.45 2.66 2.70 3.12 2.21 3.29 2.34	LYS9 SER26 SER32	3.50 2.34 2.30	ARG11	2.74	ARG227 GLN228	1.98 2.69
	HA	GLY12 ALA24	2.64 2.81	SER32 THR38	2.64 2.28	SER257	2.26	GLN228	2.51
	Other (Pi-Cat)	LYS9	3.91	LYS9	4.08				
NLRP3-NACHT domain (Chain A)	Hp	ALA98 PRO223 PHE281 ILE494 GLN495 LEU499	3.80 3.89 3.90 3.65 3.62 3.42	ALA98 PRO223 PHE281 ILE494 GLN495 LEU499	3.73 3.71 3.94 3.65 3.48 3.32			ALA98 PHE446 TYR503 THR530	3.59 3.32 3.63 3.60
	HD	GLU227 ARG449 ARG449 GLN495 SER497 GLU500 ASN527 SER529	3.23 2.36 2.35 3.28 3.16 2.85 2.82 3.15	GLU227 ARG449 ARG449 GLN495 SER497 GLU500 ASN527 SER529	3.22 2.17 2.35 3.42 3.57 2.68 2.38 3.23	ARG262 ARG262 MET523	2.56 2.12 2.65		
	HA	PRO223 PRO223 SER497 GLU500 ASP533	2.19 3.22 2.72 2.66 3.44	PRO223 PRO223 SER497 GLU500 ASP533	2.25 2.99 3.65 2.72 2.86			ASP533	2.48
IL-1β (Chain B)	Hp	VAL47 LYS55 PRO57 PRO57 MET95 VAL100	3.75 3.44 3.48 3.79 3.54 3.76	VAL47 LYS55 PRO57 MET95 VAL100	3.83 3.53 3.50 3.54 3.78	VAL3 VAL47 PRO57 VAL100 ALA115	3.67 3.77 3.70 3.59 3.80	PRO57 PRO57 LYS97 VAL100	3.84 3.61 3.78 3.43
	HD	VAL47 ASN53 LYS97 ASN102	2.32 2.39 3.19 3.16	VAL47 ASN53 LYS97 ASN102	2.38 3.04 3.89 3.80	MET95 ASN102	2.95 2.03	MET95 LYS97	2.17 2.53
	HA	GLU50 ASN102	2.71 2.49	GLU50 MET95	2.21 1.92	GLU50 LYS93 MET95	2.26 1.79 1.86	LYS92	2.16
	Other (salt bridge)			LYS97	4.23				
IKKβ (Chain C and A)	Hp	LEU11C ALA15C LEU22A LEU22C	3.99 3.72 3.75 3.74	LEU21C LEU22A LEU22C GLU23A ALA25C ILE26A	3.45 3.86 3.44 3.51 3.63 3.76	LEU18C LEU21C LEU22A LEU22C ALA25C ILE26A ILE26A ILE26A	3.82 3.80 3.66 3.76 3.95 3.32 3.70 3.82	ALA15C LEU18C LEU18C LEU18C	3.78 3.48 3.64 3.54
	HD			GLU23A GLN27A	3.39 2.00			GLU14C	2.47
SOD (Chain A and B)	Hp	LYS9A LYS9B ASN53A VAL148A	3.95 3.84 3.51 3.81	LYS9B ASN53A ASN53B VAL148A	3.90 3.35 3.73 3.91			VAL7B LYS9A ASN53A VAL148A	3.66 3.81 3.75 3.76
	HD	VAL7B LYS9A LYS9B ASN53A VAL148B	2.05 3.46 3.15 2.05 2.92	VAL7B LYS9A LYS9B ASN53A VAL148B	2.12 3.46 3.07 2.20 2.71	VAL7A VAL148A	2.06 2.13	GLY56A VAL148B	2.23 2.31
	HA	VAL7A VAL7B ASN53A	2.93 2.37 3.05	VAL7B LYS9B GLY51A	2.64 2.95 2.21	VAL148A	2.41	ASN53B THR54A VAL148B	2.22 2.34 2.52

**FIGURE 6:**
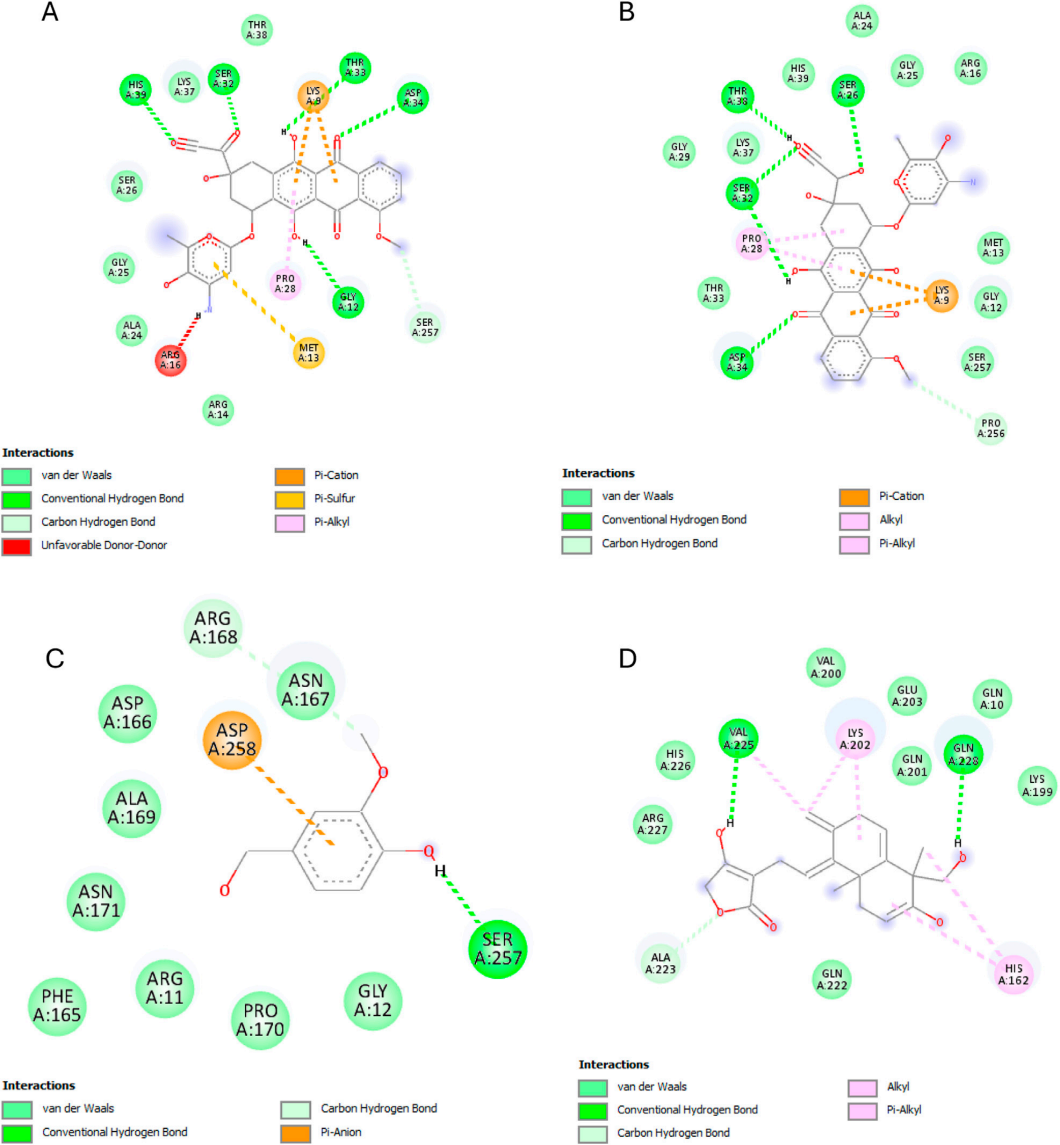
NF-кB Rel A (P65) interactions with the different ligands: **(A)** Doxorubicin, **(B)** Doxorubicinol, **(C)** Vanillyl alcohol, and **(D)** Andrographolide.

The results of this experiment show favorable binding energy between the ligands and the protein NF-кB Rel A (P65). Andrographolide also exhibited a slightly lower binding affinity (−6.951 kcal/mol) than doxorubicin, though higher than the reference ligand (−4.805 kcal/mol). The reference ligand molecule displayed fewer interactions (ASP258, ARG11, and SER257) than andrographolide which established interactions with LYS202, VAL225, and GLN 228, ARG227, and GLN228 (Figure 6).

**FIGURE 7:**
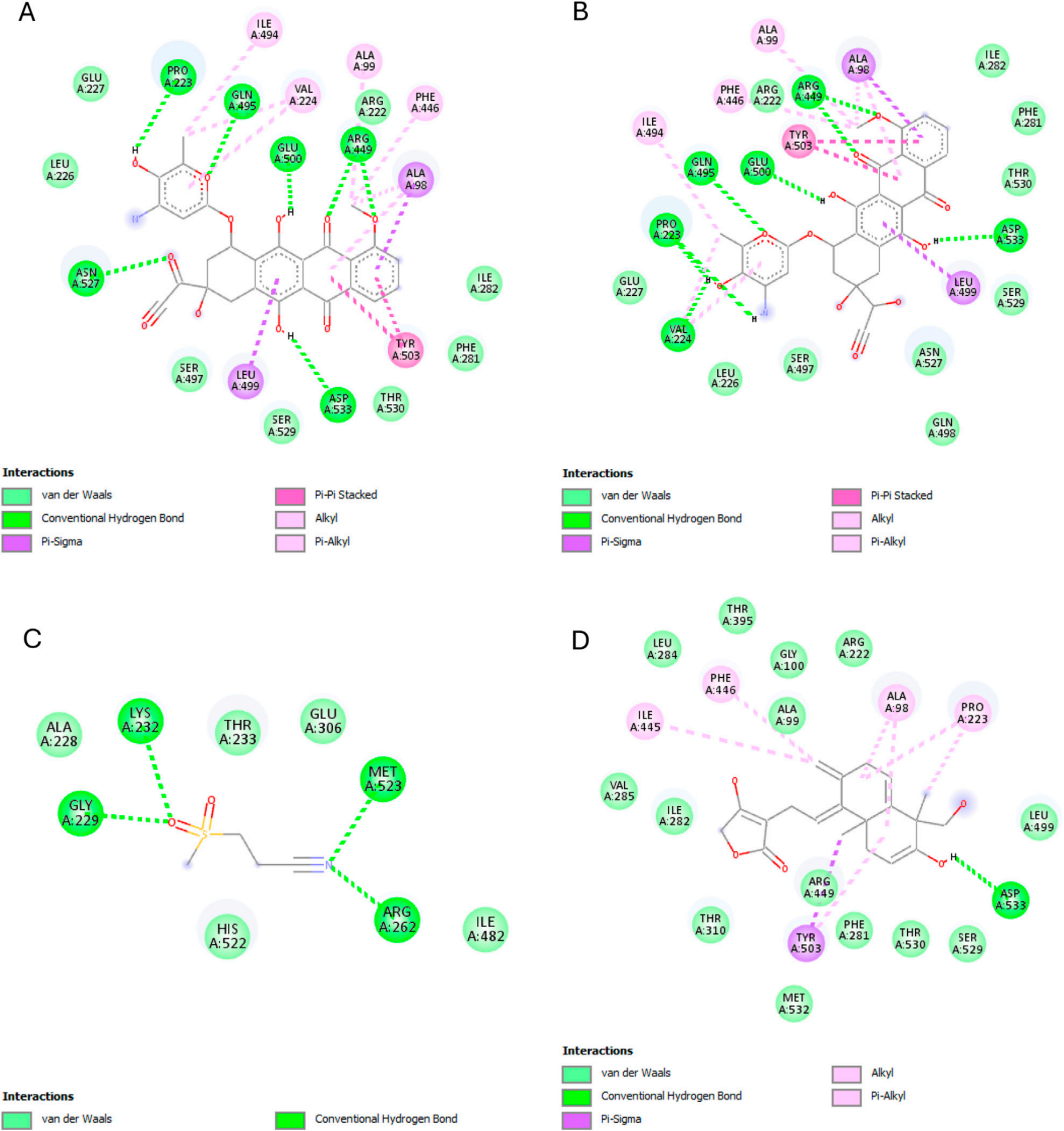
NLRP3-NACHT domain interactions with the different ligands: **(A)** Doxorubicin, **(B)** Doxorubicinol, **(C)** Dapansutrile, and **(D)** Andrographolide.

The best binding affinity (−8.223 kcal/mol) of andrographolide in this experiment was observed with the NLRP3-NACHT domain. Andrographolide displayed interactions with residues ALA98, PHE446, TYR503, THR530, and ASP533, whereas the reference ligand, Dapansutrile, only displayed hydrogen bonding with ARG 262 and MET523 (Figure 7).

**FIGURE 8:**
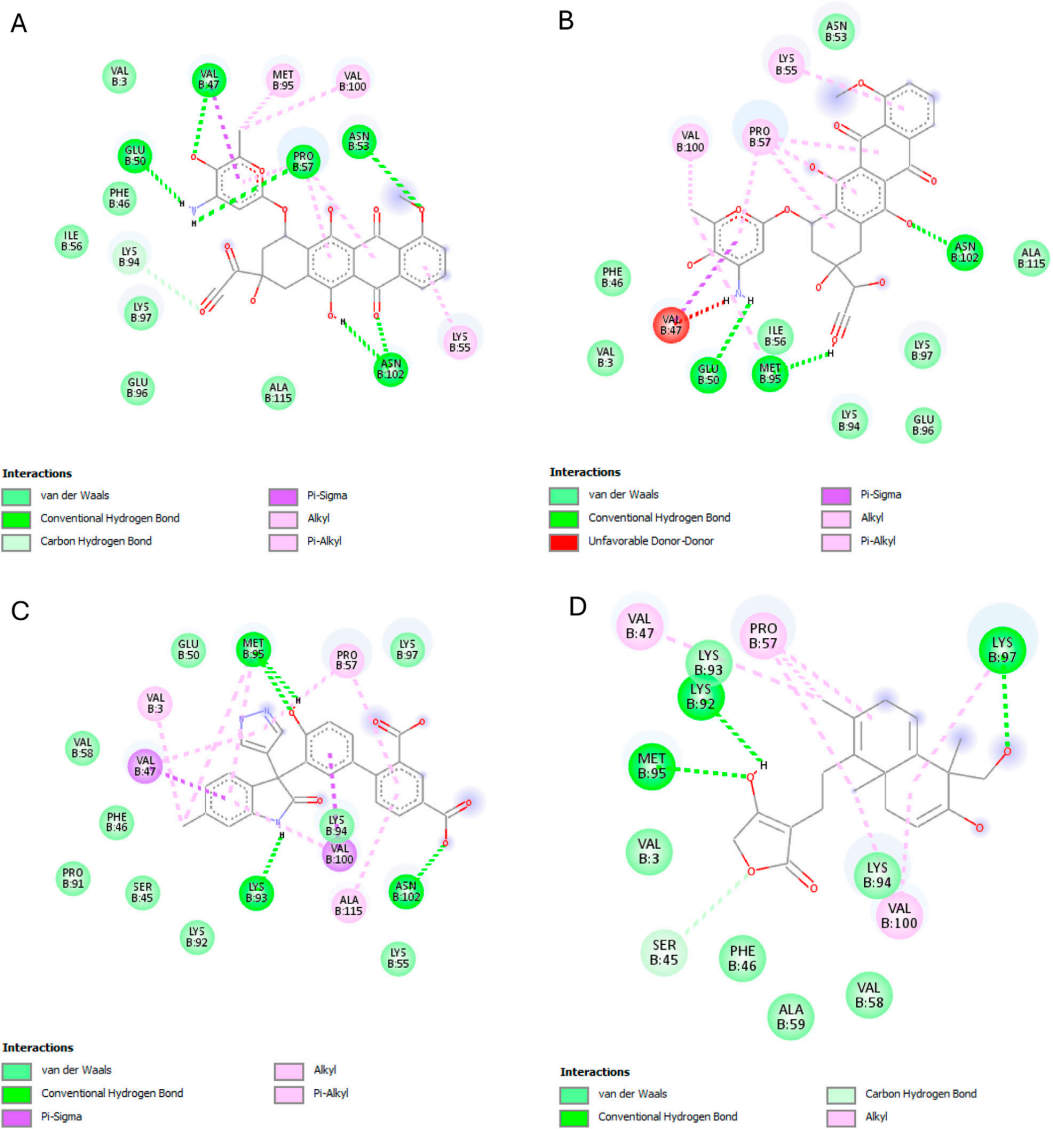
IL-1β interactions with the different ligands: **(A)** Doxorubicin, **(B)** Doxorubicinol, **(C)** (S)-2, and **(D)** Andrographolide.

Andrographolide exhibited the second-best binding affinity (−7.616 kcal/mol) with IL-1β in this experiment, though it was not lower than that of the used reference ligand (S)-2, which displayed −9.580 kcal/mol. Andrographolide exhibited interactions with PRO57, LYS97 and VAL100, MET95, LYS97, and LYS92, whereas (S)-2 exhibited interactions with VAL3, VAL47, PRO57, VAL100, ALA115, MET95, ASN102, GLU50, and LYS93 (Figure 8).

**FIGURE 9:**
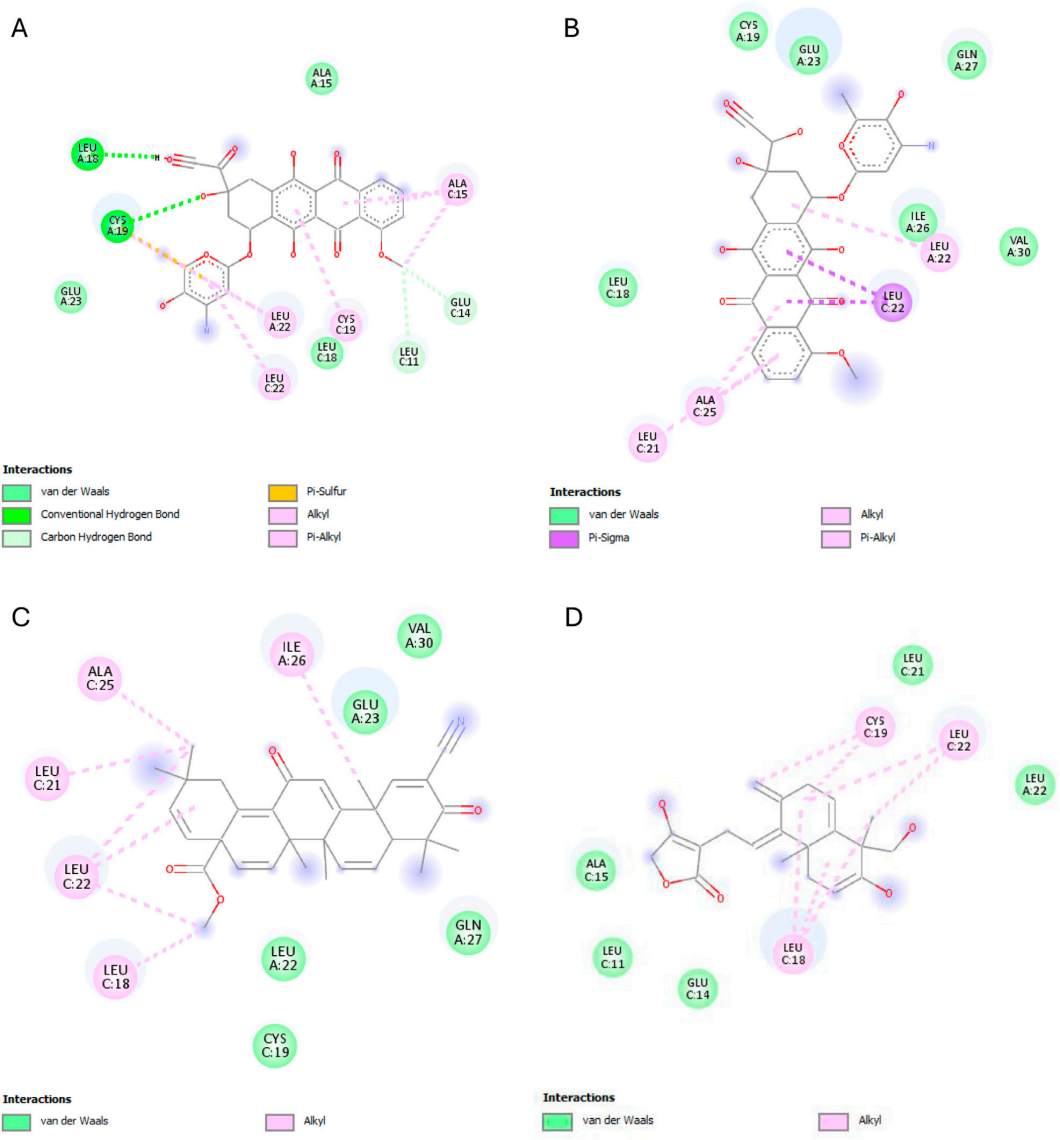
IKKβ interactions with the different ligands: **(A)** Doxorubicin, **(B)** Doxorubicinol, **(C)** MLN120B, and **(D)** Andrographolide.

IKKβ recorded the lowest overall binding energies from the four ligands, with the reference ligand, MLN120B, exhibiting the highest (−6.982 kcal/mol), followed by doxorubicinol (−5.652), andrographolide (−5.441 kcal/mol) and then, doxorubicin (−5.425 kcal/mol). Andrographolide displayed interactions with only chain C at residues ALA15C, LEU18C, and GLU14C, whereas the reference ligand, MLN120B, displayed a high binding affinity and interactions with both A and C chains at the residues LEU18C, LEU21C, LEU22A, LEU22C, ALA25C, and ILE26A (Figure 9).

**FIGURE 10:**
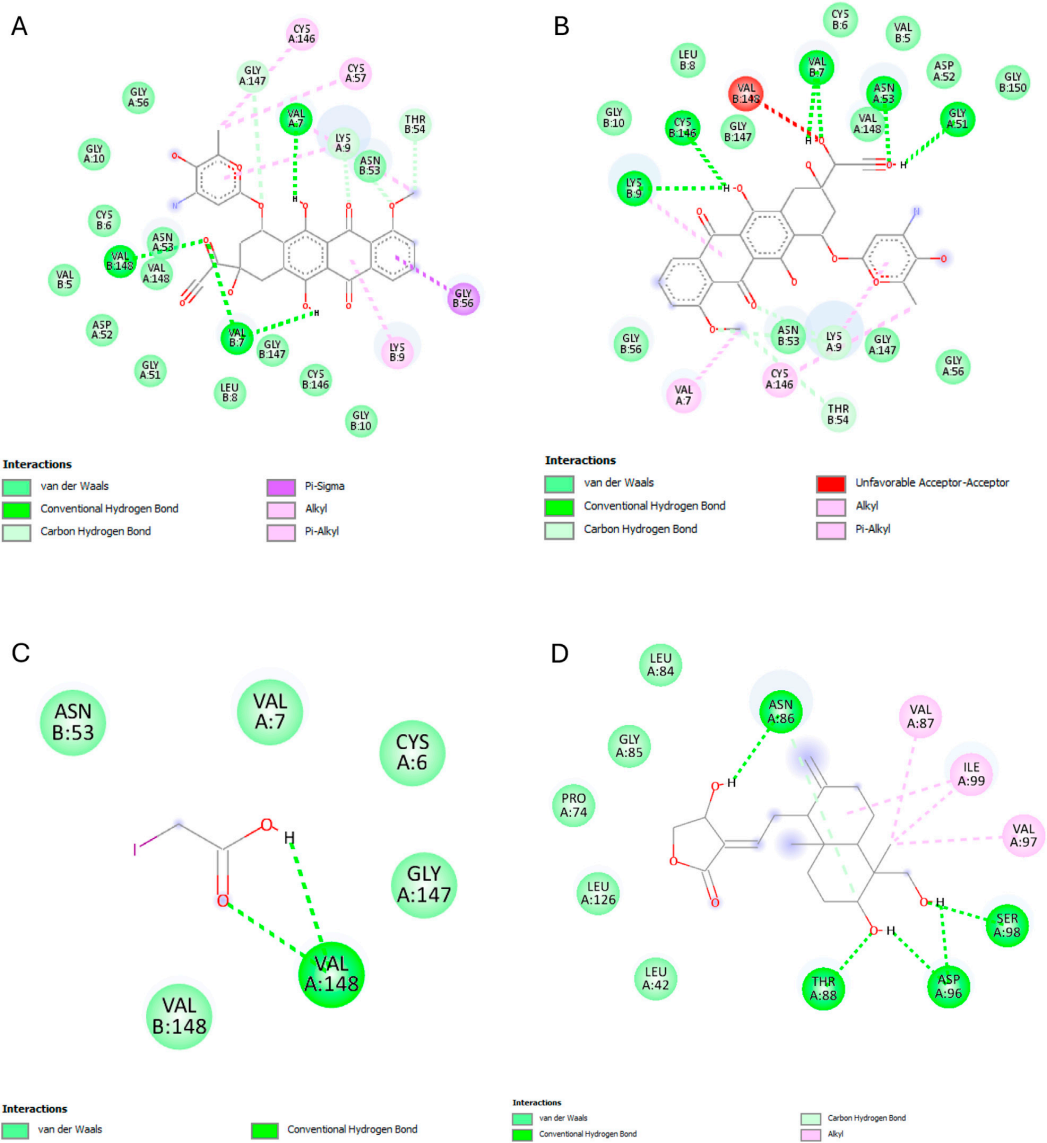
SOD interactions with the different ligands: **(A)** Doxorubicin, **(B)** Doxorubicinol, **(C)** IAA and **(D)** Andrographolide.

Most ligands displayed high binding energies with SOD, except for the reference ligand, IAA, which displayed −3.505 kcal/mol. Andrographolide displayed a −7.578 kcal/mol binding affinity, showing interactions with both the A and C chains at Residues VAL7B, LYS9A, ASN53A, VAL148A, GLY56A, VAL148B, ASN53B, THR54A, and VAL148B. Whereas IAA exhibited interactions with only chain A at VAL7A and VAL148A (Figure 10).

### 3.9 Shared binding residues and competitive inhibition patterns

Following our analysis of binding energies and interaction types that govern complex stability, we now examine the specific residues shared between doxorubicin, doxorubicinol, and andrographolide. This residue-level comparison reveals critical insights into competitive binding mechanisms that may underlie the nephroprotective effects observed experimentally. The presence or absence of common interaction sites across these ligands helps explain how andrographolide may either directly compete with DOX metabolites or exert complementary modulation of target proteins.

The molecular docking analysis reveals a complex interplay between DOX, its metabolite DOXol, and andrographolide at critical molecular targets involved in nephrotoxicity. The pattern of shared and unique interaction residues provides important mechanistic insights into andrographolide’s potential protective effects.

Starting with the NF-κB pathway, we observe that DOX and DOXol share key interactions with PRO28 (hydrophobic), SER26/SER32 (hydrogen bond donor), and LYS9 (hydrogen bond donor and pi-cation interaction) (Table 6). These interactions likely stabilize NF-κB in its active conformation, promoting transcription of pro-inflammatory genes (Karin and Ben-Neriah, 2000). Notably, andrographolide does not bind these same residues but instead interacts with ARG227 and GLN228, which are critical for NF-κB-DNA binding (Chen et al., 1998). This suggests andrographolide may inhibit NF-κB through an allosteric mechanism rather than direct competition, potentially explaining its ability to suppress inflammatory signaling without completely blocking the pathway.

**TABLE 6:** Common interactions between doxorubicin, doxorubicinol and andrographolide.

Target	Residues
NF-κB	
NLRP3	ALA98 (Hp); ASP533 (HA)
IL-1β	PRO57, VAL100 (Hp); LYS97 (HD)
IKKβ	
SOD	ASN53A, VAL148A (Hp); VAL148B (HD)

For the NLRP3 inflammasome, all three compounds interact with ALA98 (hydrophobic) and ASP533 (hydrogen bond acceptor), indicating some degree of competitive binding at these sites (Table 6). However, DOX and DOXol show additional interactions with PHE281 and ILE494, while andrographolide lacks binding to the crucial ARG449 residue involved in inflammasome assembly (Zhou et al., 2010). This partial overlap suggests andrographolide may attenuate but not completely prevent NLRP3 activation, potentially explaining its ability to reduce but not eliminate IL-1β production in experimental models.

All three ligands compete for the IL-1β active site (Table 6), sharing interactions at PRO57 (hydrophobic), VAL100 (hydrophobic), and LYS97 (hydrogen bond donor). However, DOXol uniquely forms a salt bridge with LYS97, enhancing its binding affinity (−8.112 kcal/mol) and likely stabilizing IL-1β in a pathological conformation. In contrast, AP (−7.616 kcal/mol) engages LYS97 only via hydrogen bonding, allowing it to partially displace DOX/DOXol while avoiding complete IL-1β inhibition. This competitive yet moderated interaction aligns with experimental results, where EEAP reduced IL-1β mRNA expression by 28%–31% without full suppression—a balance between mitigating inflammation and preserving essential immune function. The conserved role of LYS97 (Dinarello, 2011) underscores its importance as a therapeutic target in DOX-induced nephrotoxicity.

For IKKβ, the minimal overlap in binding residues is noteworthy. While DOX and DOXol interact with LEU22, andrographolide binds ALA15C instead (Table 6). This distinct binding pattern supports the hypothesis that andrographolide inhibits IKKβ through allosteric modulation rather than direct competition (Xia et al., 2004). The lack of shared residues here suggests andrographolide’s effects on this kinase may be complementary to its other anti-inflammatory actions.

The SOD interactions present an interesting case where partial competition may be beneficial. All three compounds interact with ASN53A and VAL148A (hydrophobic) and VAL148B (hydrogen bond donor), but only DOX/DOXol bind LYS9B (Table 5). This selective binding pattern suggests andrographolide may preserve SOD activity by avoiding interactions that would impair the enzyme’s catalytic function (Fridovich, 1995). The shared binding at VAL148 positions could allow andrographolide to partially displace DOX/DOXol while maintaining antioxidant capacity.

## 4 Discussion

One of the detrimental side effects of doxorubicin is nephrotoxicity (Afsar et al., 2020; Burke et al., 1977). Current understanding suggests that doxorubicin elevates reactive oxygen species (ROS) while depleting antioxidants, leading to oxidative stress (Arunachalam et al., 2022; Wu et al., 2021) This oxidative injury damages cells, releasing danger signals that activate inflammatory proteins such as NF-κB, NLRP3, and IL-1β. Additionally, DOX and its free radical byproducts may also directly activate these inflammatory proteins (Alasmari et al., 2022; Arunachalam et al., 2022; Wu et al., 2021). The cumulative effect of these molecular events is a decrease in renal function, which is indicated by an increase in serum urea and creatinine as well as electrolyte imbalance (Abdelrahman et al., 2020; Ikewuchi et al., 2021).

In this study, we measured SOD and TAC as oxidative stress markers, which have been used as such in closely related work (Abdelrahman et al., 2020; Antar et al., 2023). SOD, an antioxidant enzyme, was specifically selected as a target for two reasons: first, it is depleted as a result of DOX administration in many studies (Abdelrahman et al., 2020; El-Sayed et al., 2017; Hu et al., 2022); Second, it neutralizes superoxide anion radicals, the initial free radicals formed from the mitochondrial electron transport system. Superoxide anion radicals are involved in forming other radicals, such as peroxynitrite, hydroxyl radicals, and nitrogen dioxide (Maurya, 2013). Therefore, a reduction in superoxide anion radicals may decrease the formation of these other radicals. SOD converts superoxide anion free radicals to H_2_O_2_, which is then removed by CAT or GPx (Alasmari et al., 2022). TAC, on the other hand, is a primary measurement to assess the state and potential of oxidative stress in disease conditions (Maurya, 2013). Studies have shown that the administration of DOX causes a decline in SOD and TAC in the kidney tissues of rats (Abdelrahman et al., 2020; El-Sayed et al., 2017; Hu et al., 2022). Our result showed that a cumulative dose of 16 mg/kg BW administration of DOX decreased SOD by 5% and TAC by 28.20% which was not statistically significant as compared to the other studies. This suggests that oxidative injury in our model may have been in its early stage, wherein the antioxidant defense system was only minimally compromised and overt oxidative damage had not yet fully manifested. A close examination of the experimental designs in other studies with significant results for these parameters reveals that differences in the type of rats, ages, doses, routes of administration, and durations of treatment or animal experimentation may have influenced our outcome. Meanwhile, the co-administration of EEAP significantly elevated TAC (*p* < 0.05). Previous studies have shown that *A*. *paniculata* and its principal bioactive constituent, andrographolide, exhibit potent antioxidant properties. These substances have been demonstrated to significantly reduce oxidative stress across various organ systems in rodent models, including the kidneys (Mukherjee et al., 2024; Neogy et al., 2008; Owoade et al., 2022; Parthasarathy and Prince, 2023). Our results are consistent with these reports and provide evidence that EEAP can reduce DOX-induced oxidative stress and offer protection to the kidneys.

NFкB is a transcriptional factor that regulates the expression of many genes involved in inflammation (Liu et al., 2017). NFкB p65 is dependent on Toll-like receptor (TLR) senses to danger signals (from damaged or stressed cells) and is activated via the canonical pathway (Giridharan and Srinivasan, 2018). Upon activation, NF-κB p65 translocates to the nucleus and promotes the transcription of key inflammasome components, including NLRP3 and pro-interleukin-1β (pro-IL-1β), which is known as the priming step for the NLRP3 inflammasome pathway. NLRP3, on the other hand, is a Nod-like receptor (NLR) that is activated by sensing danger signals from damaged cells. In the NLRP3 inflammasome pathway, pro-IL-1β is converted to its active form IL-1β by Caspase-1 (Kaneko et al., 2019; Lopez-Castejon and Brough, 2011). This study measured the concentration of NFкB and the mRNA expressions of NLRP3 and IL-1β in kidney tissues. The result showed that the DOX treatment increased the concentration of NFкB by 23.99% (not statistically significant) and the mRNA expressions of NLRP3 and IL-1β by 128.16% and 103.96% respectively (*p* < 0.05). Our result for NLRP3 and IL-1β was consistent with another study (Wu et al., 2021). Further, the significant expression of NLRP3 and IL-1β in the DOX-only group suggests that NLRP3 may be more sensitive to subtle cellular danger signals and that NLRP3 and IL-1β could be more reliable markers for the early detection of DOX-induced inflammation. Co-administration with EEAP capsules at 125 and 250 mg significantly decreased the mRNA expressions of NLRP3 (*p* < 0.05). Our results are consistent with numerous studies demonstrating that *A*.*paniculata* possesses a strong anti-inflammatory effect (Burgos et al., 2021; Eziefule et al., 2024b; Li et al., 2022; Rahmi et al., 2022). EEAP could decrease DOX-induced inflammation in kidney tissues.

Creatinine is a waste product produced by the breakdown of creatine phosphate in muscles. Urea, on the other hand, is a nitrogen-rich substance formed in the liver as a result of protein metabolism and the urea cycle. In normal renal tissues, creatinine and urea are filtered from the bloodstream into the urine. Further, the concentration of electrolytes, which play vital roles in the biological functioning of the body (including nerve and muscle function, fluid balance, and bone health), is properly regulated by a functioning kidney. The elevation of serum creatinine and urea, as well as electrolyte imbalance, is associated with renal dysfunction (Alasmari et al., 2022; Gowda et al., 2010; Ikewuchi et al., 2021; Rosner and Bolton, 2006). Studies have shown that DOX treatment in rats causes an increase in serum creatinine, urea, and electrolyte imbalances (Alasmari et al., 2022; Gowda et al., 2010; Ikewuchi et al., 2021). Our result revealed that serum creatinine and urea in the DOX-only group increased by 20.83% and 9.98% respectively (not statistically significant). Hypernatremia was evident (*p* < 0.05), and calcium concentration increased by 12.63% (not statistically significant) in the DOX-only group. Hypernatremia and hypercalcemia were recorded in a previous study after DOX administration (Ikewuchi et al., 2021). The absence of statistically significant elevations in serum creatinine and urea levels in the DOX-only group compared to the normal control group suggests that DOX-induced nephrotoxicity was not markedly pronounced under the conditions of this study. These findings may indicate that renal impairment was at an early stage and not yet sufficient to produce measurable changes in these conventional markers of kidney function. Renal function declines by as much as 50% before a notable increase in serum creatinine level occurs (Verena et al., 2024). The non-significant alterations observed for oxidative stress parameters and NFκB (an essential transcription factor involved in the regulation of inflammation) in the DOX-only group were consistent with our findings. These findings further support the notion that doxorubicin-induced renal injury may have been minimal or confined to early-stage pathophysiological changes in this experimental model. Meanwhile, hypernatremia was reversed by EEAP.

A histopathology study was further conducted to identify histopathological changes in renal tissues. Changes identified included capsule distortion, abnormal Bowman’s space, glomerular atrophy, tubular lumen widening, and pyknosis. Some other studies have also observed similar changes in the DOX-only groups (Afsar et al., 2020; Asaad et al., 2021). The affected glomeruli and tubules contributed to a slight decline in renal function. Damaged glomeruli would lead to reduced filtration of creatinine and urea, resulting in their accumulation in the blood. Additionally, damage to the tubules, which reabsorb and secrete electrolytes, would cause electrolyte imbalance (Abd-Ellatif et al., 2022; Alasmari et al., 2022; Asaad et al., 2021; Ikewuchi et al., 2021). While we identified histopathological changes in the DOX-only group, it is important to emphasize that these changes were moderate and not severe based on our scoring system. The absence of severe histopathological changes aligns with our findings for the renal function parameters, which were not significantly altered after DOX administration. Meanwhile, co-treatment with EEAP reduced the histopathological changes observed in the DOX-only group. Our result is in agreement with another study that demonstrated that *A. paniculata* ethanolic extract improved renal function and pathological changes in renal tissues (Adeoye et al., 2019).

Having established in this study that EEAP exerts protective effects against DOX-induced nephrotoxicity, the potential interactions between andrographolide and the target proteins were subsequently investigated through molecular docking. Ligands currently being studied or in use as either agonists or antagonists for the target proteins were employed as reference ligands to determine if andrographolide exhibited superior binding to the target proteins. Additionally, doxorubicin and one of its main metabolites, doxorubicinol, were docked to compare their binding affinities with those of andrographolide.

Most of the reference ligands used exhibited poor binding energies compared to andrographolide, demonstrating that andrographolide has the potential to replace these reference ligands. This is especially true for the NLRP3-NACHT domain and SOD targets, where the binding energy of andrographolide was approximately four magnitudes better than that of the reference ligands. The binding energies exhibited by andrographolide with NF-κB (RelA), IKKβ, the NLRP3-NACHT domain, IL-1β, and SOD were −6.951, −5.441, −8.223, −7.616, and −7.578 kcal/mol, respectively. The best binding affinity of andrographolide was with the NLRP3-NACHT domain, which was comparable to the binding affinities observed during the screening of compounds in another study, which showed results ranging from −7.987 kcal/mol to −12.654 kcal/mol, although the interactions differed from those observed in this experiment (Hayat et al., 2024). The second-best binding affinity that andrographolide exhibited in this study was with IL-1β (−7.616 kcal/mol), which was lower compared to a docking study conducted on the screening of the traditional Chinese medicine database, which reported binding affinities ranging from −10.10 kcal/mol to −14.19 kcal/mol (Liu et al., 2023). For most of the target proteins, the binding energies of doxorubicin and andrographolide are comparable, although doxorubicin exhibited binding energies that were 1–2 orders ofmagnitude better. Doxorubicinol also had binding energies that were 1–2 orders ofmagnitude better than those of andrographolide. The strong binding of doxorubicin and doxorubicinol to these targets may be beneficial for fighting cancer cells but detrimental to normal cells.

A detailed analysis of the interaction patterns (Table 6) reveal that andrographolide counteracts DOX-induced nephrotoxicity through three complementary mechanisms: (1) competitive displacement at IL-1β (via shared PRO57/VAL100/LYS97 binding) and SOD (via VAL148A/B), where andrographolide’s moderate affinity (−7.616 and −7.578 kcal/mol, respectively) balances inhibition with physiological function preservation; (2) allosteric modulation of NF-κB (targeting ARG227/GLN228 instead of DOX’s PRO28/SER26) and IKKβ (ALA15C binding); and (3) partial NLRP3 inhibition (through ALA98/ASP533 engagement while sparing ARG449). This multi-target strategy, combining direct competition at select sites with nuanced regulation elsewhere, explains andrographolide’s broad-spectrum efficacy in mitigating oxidative stress without complete pathway blockade (Chao and Lin, 2010; Singha et al., 2007) and perhaps influenced the 28% TAC restoration (*p*<0.05) and reduced inflammation (52% NLRP3 mRNA reduction) in EEAP.

While these findings highlight andrographolide’s central role in the protective effects of EEAP, it is important to recognize the broader context of the extract’s therapeutic potential. This study focused exclusively on andrographolide, despite EEAP being a complex mixture containing multiple bioactive constituents. Having established andrographolide’s mechanistic contribution, the next logical step is a comprehensive characterization of the extract to identify other active compounds that may act synergistically to mitigate DOX-induced nephrotoxicity. Additionally, further investigation is needed to elucidate the mechanisms behind andrographolide’s enhanced bioavailability and potentially reduced toxicity when administered as part of the whole extract, compared to its isolated form. It is also worth noting that overt nephrotoxicity was not induced in this study; thus, conclusions are limited to the reversal of early-stage nephrotoxicity, primarily through the attenuation of oxidative stress and inflammatory responses. Future research should aim to establish a model of complete nephrotoxicity to better delineate and validate the protective effects of this extract, thereby providing a more comprehensive understanding of its therapeutic potential.

## 5 Conclusion

This study provides the first evidence that EEAP confers protection against DOX-induced nephrotoxicity, particularly in its early stages. The protective mechanism is attributed to EEAP’s dual anti-inflammatory and antioxidant activities, as demonstrated by a marked downregulation in renal NLRP3 mRNA expression and a concomitant increase in total antioxidant capacity (TAC). Histopathological assessments supported these molecular findings, revealing attenuation of the moderate pathological lesions induced by DOX. Furthermore, EEAP treatment effectively reversed DOX-induced hypernatremia, reflecting partial renal functional recovery limited to electrolyte regulation. Andrographolide, a principal bioactive compound in the extract, exhibited favorable binding affinities (approximately −5 to −8 kcal/mol) across all studied protein targets, suggesting a strong potential for interaction with key inflammatory and oxidative stress-related pathways. Collectively, these findings highlight the therapeutic promise of EEAP, particularly andrographolide, as a nephroprotective agent against DOX-induced renal toxicity.

## Data Availability

The data and materials not provided within the manuscript that support the results of this study will be made available upon reasonable request to the corresponding author.
